# Lower partial pubicectomy for postoperative complicated posterior urethral stricture

**DOI:** 10.1007/s11255-023-03746-3

**Published:** 2023-08-29

**Authors:** Xiaoming Zhang, Wei Wang, Haiyan Zhang, Lei Zhang, Chenglin Yang, Hui Zhang

**Affiliations:** 1 Department of Urology, General Hospital of Southern Theatre Command, Pepole’s Liberation Army, Guangzhou, 510010 China; 2 Medical Security Centre, General Hospital of Southern Theatre Command, Pepole’s Liberation Army, Guangzhou, 510010 China

**Keywords:** Urethroplasty, Inferior partial pubicectomy, Complex posterior urethral stricture

## Abstract

**Objective:**

To report the experience of partial inferior pubicectomy in the treatment of complex posterior urethral stricture after trauma.

**Methods:**

A total of 46 patients with post-traumatic posterior urethral stricture admitted to the Department of Urology of our Hospital from January 2013 to September 2021 were selected as the research objects and underwent urethroplasty (including nine patients who had failed previous perineal repair surgery and adopted partial inferior pubicectomy approach). Retrograde urethrograph (RUG) and urine flow measurement were performed at 1, 3, 12 and 18 months after operation, and follow-up was performed when necessary. The clinical data during treatment were statistically analyzed.

**Results:**

All 46 patients underwent urethroplasty successfully, of which nine were treated with partial pubicectomy, accounting for 19.57% of the total. The causes of the disease were motor vehicle accident in 4 cases, falling collision injury in 2 cases, and rolling injury of military exercise tank in 3 cases. Among the 9 patients, 2 were children (22.22%), aged 8 and 12 years, and 7 were adults (77.78%), aged 19–44 (28.42 ± 1.56) years. Among the 9 patients, 6 had erectile dysfunction, accounting for 66.67%. The length of posterior urinary tract stenosis was (5.12 ± 0.57) cm. The operation time was (290.34 ± 12.35) min from anesthesia induction to skin closure. Five patients received 2 U blood transfusion during operation and three patients received 3 U blood transfusion after operation. The average hospital stay was 12–16 (14.24 ± 1.25) days, and the follow-up was 12–24 (18.24 ± 1.35) months. After surgery, one patient developed HIP abscess, which was successfully treated conservatively. One patient had dysuria 1 month after operation and was successfully treated by transurethral dilatation. One case had postoperative infection and recovered after intravenous administration of potent antibiotics. Cystourethrography was performed 3 months after operation, and there was no difference between patients with wide, long or short anastomotic stretch defects. All patients met the criteria for surgical success.

**Conclusion:**

Partial inferior pubicectomy is a good surgical procedure for the repair of complicated posterior urethral stricture after operation. It is safe and reliable, can better display the prostatic apex and surgical field, shorten the length of reconstructed urethra, and has good postoperative effect. It has no direct or long-term effect on the stability of pelvis or bladder. However, further studies in a larger cohort of patients with complex posterior urethral strictures after repair are needed to demonstrate the specific indications for partial pubicectomy.

## Introduction

In urology, urethral stricture is a common disease, which is common in men. According to location of the disease, it can be divided into urinary tract stenosis before anterior urethral stricture and after, urinary tract stenosis disease because of posterior urethral stricture. The symptoms vary according to their degree, the scope and development process. The most common one is traumatic urethral stricture, the all of urethra stenosis (in which bulbous urethral stricture accounts for 50.00%), posterior urethral stenosis (stricture accounts for 40.0%), anterior urethral stenosis (stricture accounts for 10.00%) [1]. Repair of congenital or acquired injuries is difficult because the posterior urethral lesions are deeply located in the pelvis behind. Although some surgical techniques have been advocated and introduced into the treatment of complex posterior urethral stricture, such as perineal approach and alternative approach, according to a search of the previous literature [1] pubic [2] subpubic excision [3] after the pubic [7, 8] and a retroactive approach of the rectum [4], there are still less than 5% of cases are due to relevant limiting factors (urethral shortage, narrow intraoperative visual field stricture, difficulty in exposure and anastomosis, intraoperative bleeding, urinary incontinence, erectile dysfunction, postoperative restenosis, and inadequate urethral mobility [5] and so on) cannot achieve the desired therapeutic effect, and even the possibility of disability. Golimbu etc. [6], After treatment with the pubic approach in 287 patients, Golimbu et al. [6] found that partial pubic resection provided comparable equivalent exposure and fewer complications than to total pubic resection, and the complications were lower than the latter, which made it an alternative to the standard approach for some patients with complicated posterior urethral strictures with difficult access stricture [3, 9 and 10]. In this study, 46 patients with complex posterior urethral stricture admitted to the department of urology of our hospital from January 2013 to September 2021 were analyzed retrospectively, to explore and the safety and effectiveness of lower partial pubic resection. The report was described as follows:

## Materials data and methods

### General data

Sample source: urology department, our hospital (January 2013 to September 2021), sample size: 46 patients with complex posterior urinary tract stenosis. The inclusion criteria were as follows: Inclusion criteria—(1) preoperative RUG diagnosis of stenosis ≥ 2 strictures lanes (or their length 2 cm); (2) patients with urinary tract infection and their condition can be effectively controlled by clinical treatment; Exclusion criteria—(1) patients with preoperative RUG diagnosis of stenosis < 2 channels (or its length < 2 cm); (2) patients with urinary tract infection who have not been cured; (3) Anterior and posterior urinary atresia and urethral anastomosis cannot be completed due to pelvic bone fragments. According to the above inclusion and exclusion criteria, forty-six patients with complex posterior urinary tract stenosis were included in the study. All of them were stricture, all male, aged 21–75 (37.4 ± 4 5.67) years for adults and 5–15 (13.24 ± 24 1.54) years for children, were enrolled in the study. There were 16 cases of complicated posterior urinary tract stenosis stricture caused by pelvic fracture caused by car accident, and 23 cases of complicated posterior urinary tract stricture caused by crushing pelvis with heavy objects and seven cases of complicated posterior urinary tract stenosis stricture caused by iatrogenic injury, respectively. The average length of posterior urinary tract stenosis was (3.15 ± 0.67) cm. RUG is the main diagnostic method was RUG, supplemented by urethroscopy and urodynamic examination. Joining the group, they all patients signed the informed consent form (voluntary), and all the data were complete and valid, which was approved by the Medical ethics Committee of our hospital.

### Surgical method

#### Patient’s position

**Fig. 1:**
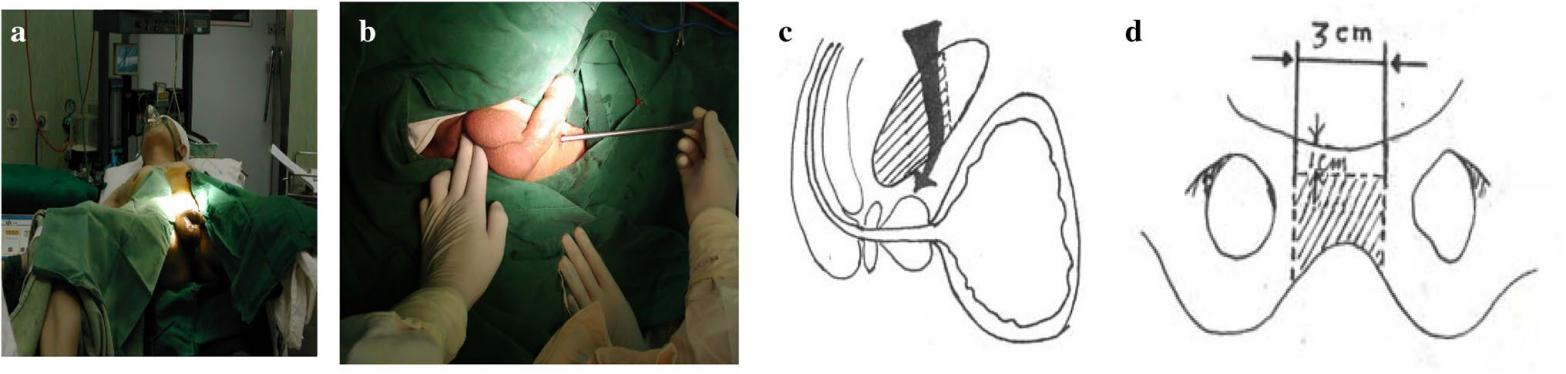
Lithotomy position, impact test, and boundary of the excised pubic bone symphysis excision

After general anesthesia, the lithotomy position was taken, abduct both lower limbs, and carry out routine disinfection with towel. Transurethral and transvesicostomy urethroscopy or flexible cystoscopy were performed to obtain the specific location, degree and length of the complex posterior urinary tract stenosis, and the imaging data posterior urinary tract stones calculi and embedded bone fragments were obtained. In the complex posterior urethral stricture, fill the ribbon gauze impregnated with iodophor into the rectum to prevent contamination pollution (as shown in Fig. 1a).

#### Incision

**Fig. 2:**
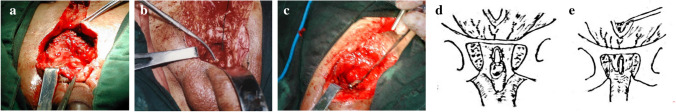
Subpubic partial resection, exposure of posterior urethral stricture, resection of urethral stricture, and anastomosis of the new urethra to the posterior urethra

Preoperative impact test to determine the degree of urethral stricture (Fig. 1b). The bulbourethra was cut down as a narrow segment through the midline and *λ*-shaped perineal incision. If the scar tissue extends above the suprapubic prostate, a subumbilical midline incision is made to create an abdominal combined perineal exposure. An exposure is formed through the median incision under the umbilicus. Using periosteal lifter to remove elevator, the attached abdominal muscles on the external surface of the pubic bone were removed 1.5 cm on both sides of the pubic symphysis, leaving about 1 cm of the pubic bone. Use a chisel to remove the suprapubic bone. The wedge bone was removed from the upper surface of the pubic bone using a bone chisel (Figs. 1c, d,  2a, b).

#### Urethral exploration and repair

**Fig. 3:**
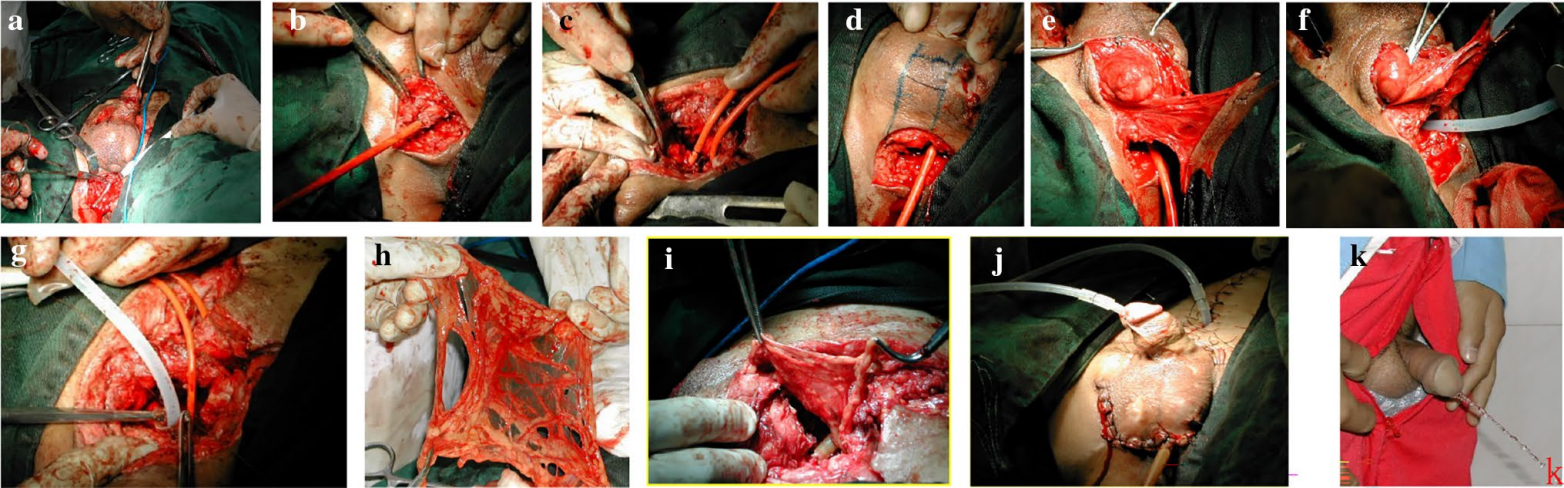
Surgical Steps. Liberation of bulbar urethra (**a**, **b**); Lower body incision (**c**); Urethroplasty with diaphragm flap (**d**–**f**); Anastomosis of new urethra and prostate urethra (**g**); The greater omentum fills infrapubic space (**h**, **i**); Place the bracket (**j**); The patient urinates after operation (**k**)

The depth of the osteotomy varies according to the required exposure. Peel the prostate from the retropubic callus, and be careful to avoid dissection in peeling at the posterior plane of the prostate (Fig. 2c–e). After the formation of retropubic space, if there are any complications, a rectourethral fistula resection will be performed. Fistula core was opened into the rectum and excised. The urethral and rectal ends are closed in two layers. Then open the bladder through the cystostomy and check whether there is scar on the bladder neck. Scars are removed until healthy, soft tissue is reached. If this means destroying the bladder neck, at the same time, the proximal end is built to provide self-control. Then move the bulbourethral from the distal end of the perineal membrane, usually reaching but not exceeding the scrotal boundary. There are two options for completing a tension-free anastomosis. If the length of the anterior urethra is sufficient, the anterior urethra passes through the normal subpubic access. Infrapubic pathway, if chordee (penile erection and bending) is caused through the infrapubic path, the calf will be separated at the midline and rerouted. A diverted tension-free end-to-end mucosal–mucosal urethral anastomosis was performed with 4–0 polylactic acid through in Foley catheter (14 F for children and 20 F for adults). Insertion of suprapubic catheter was inserted through the bladder vault. Secure the bladder to the pubic bone on both sides with non-absorbable polypropylene suture (No.1). To eliminate the dead space around the anastomosis, the pedicled omentum was removed from the abdominal cavity and slid into the pelvis. Take levator testis muscle flap or Dato muscle flap was taken from the scrotum to wrap around the urethral anastomosis. Suspension ligament was requiring supraurethral redirection. Suprapubic tubes were placed to drain the urinary tract, and two suction tubes were placed in the retropubic space. Place the suction tubes and anatomically close it. The specific operation steps are shown in Fig. 3.

#### Postoperative evaluation

The suction tube was removed on the 3rd postoperative day after operation. The fifth day after operation, the patient was discharged after removal of the catheter and suprapubic canal. RUG test was performed after 3 weeks, and the catheter was removed if the postoperative healing was good with no extravasation. The catheter was then removed. Subsequently, the suprapubic tube is clamped to give the patient a chance to urinate. If urination was free, remove the suprapubic canal tube. Follow-up time: RUG and urine flow measurements and urography were measured at 1, 3, 12 and 18 months after operation, respectively, and micturition cystourethrography was performed at 3 months after operation. The definition of successful operation: the patient’s subjective urine flow is good, and there is no residual urine after urination. The maximum flow rate of adults is 15 ml/s in adults, and the maximum flow rate of children is 12 ml/s. RUG diagnosis. Surgical failure was diagnosed that urethral caliber is normal and smooth. Definition of operation failure: A variety of postoperative auxiliary procedures operations are needed, such as dilatation, endoscopic urethrotomy or repeated open urethroplasty. RUG was used to diagnosed abnormal urethral diameter and unsmooth urethral caliber. Symptom evaluation: Ask directly about urine flow, incontinence and erection was performed with the consent of patient’s consent.

## Results

According to statistics, 9 out of 46 were treated with partial inferior pubicectomy, accounting for 19.57% of the total. The causes of the disease were motor vehicle accidents in four cases, falling collision injuries in two cases, and tank crushing injuries in military exercises in three cases. All patients were placed with suprapubic tubes at the initial trauma. Among the 9 patients, there were two children, accounting for 22.22% (2/9), aged 9 and 12, respectively. Seven patients were adults, accounting for 77.78% (7/9), aged from 19 to 44 (28.42 ± 42.56) years. Among the nine patients, six had erectile dysfunction, accounting for 66.67% (6/9), which may be related to the initial trauma. The length of posterior urethral stricture was (5.12 ± 0.57) cm. Operation time: (290.34 ± 12.35) min from the induction of anesthesia, five patients received 2 U during operation and patients received 3 U blood transfusion after operation. hospitalization time was 12 ~ 16 (14.24 ± 24 1.25) day. After operation, one patient developed hip abscess, by conservative treatment. Table 1 shows the data of nine patients. Partial pubicectomy did not differ between adult and pediatric patients.

**Table 1:** Surgical operation and postoperative data of nine patients treated by lower pubicectomy

Serial number	Age (years old)	Postoperative follow-up of erection after previous surgery	Function defect (cm)^a^	Operation (b) times)^b^	Time (min)	Transfuse blood (U)^c^	Qmax (mL/s)	follow-up time (month)
1	8	Yes	4	2	300	2	13	24
2	12	Yes	4	2	310	2	14	28
3	19	Yes	5	2	280	3	10	24
4	28	Yes	6	2	300	2	15	12
5	33	Yes	5	3	330	3	17	18
6	44	Yes	5	2	290	3	19	7
7	30	Yes	4	3	330	3	15	32
8	29	No	4	2	280	2	17	18
9	27	Yes	5	3	290	3	5	24

*max* maximum flow, *PFPUS* posterior urethral stricture after traumatic pelvic fracture after trauma

^a^Length of urethral stricture measured during operation, ^b^Previous perineal urethroplasty to correct posterior urethral stricture corrected by perineal urethroplasty, ^c^Blood transfusion in unit (each unit (U) is equivalent to 200 cc of blood)

In patients with previous perineal repair, the anatomy is more difficult for patients who have been repaired by perineum in the past. No urinary tract infection and fistula formation were found after operation. There was no difference in the appearance of pubic bone between appearance before and postoperative. Urination was completely normal in all patients. After operation, all patients urinated completely normal. The follow-up period was 12 to ~ 24 (18.24 ± 24 1.35) months. All the nine patients met the criteria of successful operation except one patient who had dysuria at 1 month after operation and was successfully treated by urethral dilatation. One case of postoperative infection and recovered after intravenous administration of potent antibiotics. Urinary cystourethrography was performed 3 months after operation, and it was found that there was no difference in the results between patients with wide, long or short anastomosis distraction defect. There is no difference in the results between adult and pediatric patients.

## Discussion

The diagnosis and treatment should be based on the history, signs, urethral instrument examination and urethra. At present, with the increase of posterior urethral stricture, traditional surgical methods can no longer meet its clinical needs. In addition, the traditional operation is difficult, and there are many postoperative complications. And the posterior urethral stricture has a special location (long length, high position, false path, serious pelvic deformity or urethrorectal fistula, history of complicated factors such as urethral rectocele fistula and multiple operations, etc. [11] (Complicated surgical history). For a long time, the treatment of complex posterior urethral stricture has always puzzled urologists and is also a difficult point in clinical treatment. However, the past clinical practice shows that a single surgical approach and method is difficult to achieve the ideal therapeutic effect for the clinical treatment of complex post-traumatic, urinary tract stenosis after, the surgery way stricture. Only by scientifically reasonably choosing a surgical method with little trauma and high success rate according to the patient's specific condition of the performer patient and the experience of small trauma, high success rate of the operator therapeutic purpose can be achieved. In addition, combined with the success rate is influenced affected by many factors, and the individualization of the surgical method is extremely critical. It is reported in literature that the success rate of different surgical approaches and methods (78–97%) [11] is relatively high.

Through the search, it is found that there are many surgical methods for the treatment of complex post-traumatic urinary tract stenosis after trauma in clinical medicine, with different advantages and disadvantages. Among them, perineal approach and pubic approach are relatively common and multi-purpose. The former is easy to simple, minimally invasive and has low complications [12]. Although the latter approach is complicated and traumatic, it can fully expose the proximal end of posterior urethral stricture and improve the success rate of urethral anastomosis [13], which is two basic ways of open surgery. The perineum is located above the outlet of the pelvic outlet, and its deep boundary is the pubic arch and anterior pubic ligament, and the lateral side of the pubic inferior branch, the sciatic branch, the ischial tuberosity, tubercle and the lateral side of the sacral tubercle ligament. The space in these boundaries is partially diamond shaped and rhombic, which is divided into genitourinary triangle and anal triangle by the line extending between the two ischial tubercles. The bottom of the perineum forms is pyramid shaped and the top is formed by the apex of the prostate gland. If you want to reach the apex of the prostate, you should first enter the base of the pyramid-shaped bottom (through the perineum) or remove the lower part of the perineum before reaching pubic part, and then you can directly reach prostate. Forty-six patients with complicated posterior complex post-traumatic urinary tract stenosis after trauma stricture group, and about 80.43% (37/46) of the patients were treated by perineal approach. The operation was successful and the curative effect was satisfactory. The remaining nine patients (19.57%) who had failed of perineal repair surgery. Combined with the specific conditions of these nine patients, most of them were patients with changed or in the behavior of the part of the posterior urinary tract, and were failed in the treatment of perineal repair. The previous literature was searched [14] and combining with the practice in this paper, it is found that because cutting off part of the lower edge of pubic margin bone will not destroy the stability of the pelvis and the bed is relatively small, the safety is high. The initial treatment of complicated posttraumatic complex post-traumatic urethral stricture only involves suprapubic cystostomy, which may have adverse effects on the subsequent repair of urethral strictures with extensive fibrosis stricture. Because of the retropubic space may occur because of its failure to expel perivesical and perineal hematoma, retropubic space may have extensive fibrosis. If the urethral reduction is not sufficient, the urethral end may be opened, resulting in complex posterior urethral stricture. However, partial pubicectomy approach provides surgeons with the feasibility of extensive reconstruction with one operation instead of operations. However, it should be paid attention to note that some defects in some lower pudendal resection approaches. For example, 66.67% of the nine patients admitted in this paper suffered from erectile dysfunction due to preoperative trauma, and the author could not evaluate the effect of partial pubicectomy on their erectile function. Another patient could not be evaluated because he was only 8 years old. The only adult patient has the ability before surgery remained is still the same after surgery. The 12-year-old didn’t have an erection, but after one year, he had gained some strength. And most importantly, all patients have no urinary incontinence. In contrast, in the perineal approach, the proximal urethra can be seen deep in the depths of the pelvis. Although it is difficult to suture cases, but as a part of the surgical field of the operation of some infravaginal approaches and both ends of the urethra can be seen well, so the suture effect is better and the follow-up effect is very good, low risk. The probability of erectile dysfunction is extremely low, accounting for 5.41% (2/37), but the overall surgical success rate of the operation is only 70.27% (26/37), which may be due to the fact that the pubic approach is too deep and narrow to allow the use of surgical instruments, and is not completely related to the exposure of the posterior pubic space. On the contrary, sympathectomy can expose the advantage of exposing a wider field of view. Larger visual field, but whether it is suitable for the treatment of complex post-traumatic urinary tract stenosis after trauma is still controversial. Because complete (partial) resection or incision of the pubic bone can fully expose the posterior urethra and the distal end of bladder neck, but the stability of the pubic bone may be affected, which may lead to chronic pain and gait disorder. Although many reports have confirmed the advantages and relatively few complications of the combined transpubic and pubic-perineal approach, total pubic resection is recommended [17, 18] which has not been accepted as a standard procedure by urologists at home and abroad as a standard operation, nor has it been recommended clinically for in the past twenty years. Tension-free urethral anastomosis was achieved by primary perineal repair of complicated post-traumatic urethral stricture, including further circumferential mobilization of penile suspension penis suspensory ligament, separation of proximal body, resection of inferior pubic resection, bone and urethral diversion around the body. Eight patients of complex posterior urethral distraction defect were treated and the results were satisfactory with good results. Because through further urethral mobilization, healthy urethra can often be stretched by1 ~ 2 cm. If the stoma is still under a certain tension due to the high position of the prostate, the proximal end will begin to separate from calf to the distal end about 4 to 4 ~ 5 cm distal along the relatively avascular midline plane, achieving a significant urethral extension and at least 1 ~ 3 cm of urethra can be obviously prolonged. At the same time, when the wedge resection of pubic bone is wedge shaped and the space around the posterior urethra is fully exposed, it is shortened by 1 ~ 3 cm, and prostate urethra is improved, which is convenient for surgical repair. We dissociate the moving urethra between the sectioning bodies to septa, and create a channel through the inferior surface, allowing a significantly so that the obviously shorter and more direct route to can reach the prostatic urethra for anastomosis. In eight cases, after achieving anastomosis with such a bone resection, it was found that there was no need to wrap the urethra around the body to provide extra length. After anastomosis through such bone resection. The clinical outcome was satisfactory in results of all cases were satisfactory. In addition, the author also found that a single perineal incision can remove part of the lower pubic bone removed through single perineum part and large urethral activity, which can solve the complex defect of complex adult posterior urethral stricture defect defects, exceeding 3 cm and reduce the surgical trauma caused by pubic-perineal approach. If this kind of posterior urethral surgery can performed through pubic approach. However, it must be emphasized that when replacement urethroplasty is needed [19, 20] this. The approach will have some limitations on the graft. Otherwise, replacement tissue such as the scrotal valve and other tissues should be used instead. Long-term follow-up is strongly recommended to better evaluate and explain indications and contraindications.

## Conclusion

Partial pubic excision is a good surgical procedure to repair complex posterior urethral stricture after a good operation, which is safe and reliable, and can better display the patient’s tip of the prostate and operating field of patients, shortens the reconstruction urethral length of reconstructed urethra, has good postoperative effect, has no direct or long-term influence on the stability of the pelvis or bladder, and has the advantages of less surgical trauma and fewer postoperative complications. However, it is necessary to further study the patients with complex posterior urethral stricture in a larger cohort to prove the specific indications of partial pubicectomy.

## Data Availability

The experimental data used to support the findings of this study are available from the corresponding author upon request.

## References

[1] Shenfeld OZ, Gdor J, Katz R (2008). Urethroplasty, by perineal approach, for bulbar and membranous urethral strictures in children and adolescents. Urology.

[2] Pratap A, Agrawal CS, Tiwari A (2006). Complex posterior urethral disruptions: management by combined abdominal transpubic perineal urethroplasty. J Urol.

[3] Andrich DE, Mundy AR (2008). What is the best technique for urethroplasty?. Eur Urol.

[4] Abdalla MA (2008). A posterior sagittal pararectal approach for repair of posterior urethral distraction injuries. Eur Does Urol.

[5] Webster GD, Guralnick ML (2002). Reconstruction of posterior urethral disruption. Urol Clin.

[6] Golimbu M, Al-Askair S, Morales P (1990). Transpubic approach for lower urinary tract surgery: a 15-years experience. J Urol.

[7] Middleton, Anthony W (1977). A comparison of the morbidity associated with radical retropubic prostatectomy with and without pubectomy. J. Urol..

[8] Takenaka A, Tewari AK, Leung RA (2007). Preservation of the puboprostatic collar and puboperineoplasty for early rrcovery of urinary continence after robotic prostatectomy: anatomic basis and preliminary outcomes. Eur Urol.

[9] Gupta NP, Mishra S, Dogra PN (2008). Does a previous end-to-end urethroplasty alter the results of redo end-to-end urethroplasty in patients with traumatic posterior urethral strictures?. Int J Urol.

[10] Orabi S, Badawy H, Saad A, Youssef M, Hanno A (2008). Post-traumatic posterior urethral stricture in children: how to achieve a successful repair. J Pediatr Urol.

[11] Thibaut C, Laurent BG (2007). Anastomotic ur ethr oplasty for posttraumatic urethral stricture: previous urethral manipulation has an egative impact on the final outcome[J]. J Urol.

[12] Sedy J, Nanka O, Belisova M (2006). sulcus nervi dorsalis penis/clitoridis: anatomic structure and clinical significance. Eur Urol.

[13] Wang Z, Zhenxiang L, Yin Z et al (2015) Analysis of 51 Cases Improved Anal Stapled Transanal Rectal Resection(STARR) Combined pubic rectum muscle of drug release in the treatment of outlet obstructive constipation effect. J Liaoning Univ Tradit Chin Med 3:169–171

[14] Ghoniem G, Elmissiry M, Weiss E (2008). Transperineal repair of Complex Rectourethral Fistula complex rectourethral fistula using gracilis muscle flap interposition—can urinary and bowel functions be preserved?. J Urol.

[15] Cooperberg MR, McAninch JW, Alsikafi NF, Elliott SP (2007). Urethral reconstruction for traumatic posterior urethral disruption: outcomes of a 25-year experience. J Urol.

[16] Kizer WS, Armenakas NA, Brandes SB, Cavalcanti AG, Santucci RA (2007). Simplified reconstruction of posterior urethral disruption defects: limited role of supracrural rerouting. J Urol.

[17] Zhang J, Xu YM, Qiao Y (2006). An evaluation of surgical approaches for posterior urethral distraction defects in boys. J Urol.

[18] Rourke KF, McCammon KA, Sumfest JM (1818). Open reconstruction of pediatric and adolescent urethral strictures: long-term followup. J Urol.

[19] El-Sheikh MG, Ziada AM, Sadek SZ, Shoukry I (2008). Pediatric and adolescent transperineal anastomotic urethroplasty. J Pediatr Urol.

[20] Singla M, Jha MS, Muruganandam K (2008). Posttraumatic posterior urethral strictures in children-management and intermediate-term follow-up in tertiary care center. Urology.

